# Lactic Acid Bacteria for Delivery of Endogenous or Engineered Therapeutic Molecules

**DOI:** 10.3389/fmicb.2018.01821

**Published:** 2018-08-03

**Authors:** Peter A. Bron, Michiel Kleerebezem

**Affiliations:** ^1^NIZO Food Research BV, Ede, Netherlands; ^2^BE-Basic Foundation, Delft, Netherlands; ^3^Host-Microbe Interactomics Group, Department of Animal Sciences, Wageningen University & Research, Wageningen, Netherlands

**Keywords:** lactic acid bacteria, probiotics, *Lactococcus lactis*, *Lactobacillus*, genetic engineering, therapeutic molecules

## Abstract

Food-grade lactic acid bacteria (LAB) are considered suitable vehicles for the production and/or delivery of health promoting or therapeutic, bioactive molecules. The molecules considered for health-beneficial use include the endogenous effector molecules produced by probiotics (mostly lactobacilli), as well as heterologous bioactives that can be produced in LAB by genetic engineering (mostly using lactococci). Both strategies aim to deliver appropriate dosages of specific bioactive molecules to the site of action. This review uses specific examples of both strategies to illustrate the different avenues of research involved in these applications as well as their translation to human health-promoting applications. These examples pinpoint that despite the promising perspectives of these approaches, the evidence for their effective applications in human populations is lagging behind.

## Lab for Food-Grade Bioactive Delivery

Lactic acid bacteria (LAB) have a long history of safe consumption in the form of fermented food products and as a consequence are considered food-grade. LAB encompass various bacterial genera, including *Lactococcus*, *Streptococcus*, *Lactobacillus*, *Leuconostoc*, and *Pediococcus*. The safety status of these bacteria has inspired their use for the production and delivery of bioactive molecules that intend to improve consumers’ health. An important concept in this domain is the use of probiotics that are live microorganisms that when administered in adequate amounts confer a health benefit on the host (Hill et al., 2014), a domain dominated by species of the *Lactobacillus* genus. An alternative approach employs LAB for the engineered production and/or delivery of heterologous bioactive molecules, which mostly employs *Lactococcus lactis* as a production host, because of its extensive genetic toolbox. Both conceptual approaches are illustrated in this review through specific examples that in our opinion are closest to actual application as health promoting concepts. This highlights the impressive potential of using these bacteria for bioactive delivery, but also exemplifies the complexity of translation toward predictable effects in human applications.

## The Probiotic Lactobacilli

*Lactobacillus* species encompass a group of LAB that are encountered in a diverse range of nutrition-rich environments, including the human and animal gastrointestinal tract, plants, as well as food and feed (Duar et al., 2017). Specific *Lactobacillus* strains are marketed as probiotics.

An intrinsic characteristic of these probiotics lactobacilli is that they produce and deliver (specific) health promoting effector molecules *in situ* that modulate host physiology in a health-promoting direction. For many probiotic lactobacilli the molecular mechanism by which they impact host health remains largely unknown. However, in depth molecular research on specific probiotic strains has generated an impressive knowledgebase of the effector molecules of these strains that play a role in the modulation of specific host pathways. Many of the probiotic effector molecules that have been described appear to reside in the bacterial cell-envelope (Bron et al., 2011, 2013; Lebeer et al., 2018). Since the research related to probiotic effector molecule discovery has recently been reviewed, we do not expand this section beyond the exemplary case discussed below. The chosen example is in our opinion among the best-described in this field, and includes technological improvements by encapsulation for *in situ* delivery of the probiotic bacteria and/or their effector molecules (Li et al., 2016).

The major secreted proteins P40 and P75 produced by the extensively studied probiotic strain *Lactobacillus rhamnosus* GG were demonstrated to modulate epidermal growth factor receptor (EGF-R) activity in epithelial cells, thereby modulating Akt activation. This interaction was shown to inhibit cytokine-induced apoptosis in epithelial cells *in vitro*, and to promote epithelial cell growth *ex vivo* in human and mouse colon-tissue explants (Yan et al., 2011; Yan and Polk, 2012). Importantly, EGF-R modulation by P40 protected mice against chemically induced colitis (Yan et al., 2011; Yan and Polk, 2012). Notably, homologues of these two secreted proteins are present in other *L. rhamnosus* strains as well as in the related species *Lactobacillus casei*, implying that the effects reported for *L. rhamnosus* GG could potentially also be achieved with related strains (Bauerl et al., 2010). The same effector molecules probably underlie the observation that *L. rhamnosus* GG is able to stimulate epithelial wound healing in *in vitro* scratch assays using both skin- and gingival- human epithelial-cell lines (Mohammedsaeed et al., 2015; Fernandez-Gutierrez et al., 2017). Interestingly, *in vivo* transcriptome responses in human duodenal tissues following *L. rhamnosus* GG consumption revealed significant modulation of epithelial wound healing pathways in the human intestinal mucosa (van Baarlen et al., 2011), indicating the congruency of these molecular interactions in different model systems. However, the connection of these conserved responses to reliable health benefits in human populations remains highly challenging. This may be due to the extensive degree of human individuality and the corresponding individualized physiological relevance of such cellular modulations, which could predominantly depend on the baseline molecular state of an individual. This conceptual view was previously coined as the “band-width of health” and may represent a principal hurdle in the reliable application of probiotics and/or their effector molecules in human (Bron et al., 2011; van Baarlen et al., 2011; Klaenhammer et al., 2012).

## Engineered Lactococci

For specific diseases it is well established which human proteins are underrepresented in the diseased population and/or have an established role in therapy. LAB engineered to produce such heterologous bioactive and/or therapeutic molecules provide an approach to delivering such molecules *in situ* as live bacterial therapeutics, or may serve as a production host to obtain these molecules in purified form. General genetic engineering capacities and in particular the controlled gene expression toolboxes available for the lactobacilli are lagging behind those that have been developed for the paradigm LAB *L. lactis* (Kok et al., 2017). As a consequence, the majority of these engineering approaches for the production (and delivery) have employed *L. lactis* as a production host. A lot of effort has been invested in the production and delivery of vaccine-antigens and allergens by engineered lactococci, and since this area has recently been reviewed (Wyszynska et al., 2015; Wang et al., 2016; Szatraj et al., 2017) we focus on some specific examples of alternative categories of bioactive molecules, including bacterial enzymes and human factors known to modulate mucosal physiology and/or immune function. The examples presented were chosen on basis of available scale-up and commercialization strategies and employ *L. lactis* as a production host.

## Lactococcal Production of an Antimicrobial Enzyme

*Staphylococcus simulans* harbors *lss* which encodes a 25 kDa antibacterial protein termed lysostaphin that hydrolyzes the Gly–Gly bond present in the cell wall of *Staphylococcus aureus*, thereby lysing this important pathogen (Thumm and Gotz, 1997). Lysostaphin administration in a nasal spray effectively reduces *S. aureus* carriage, supporting the prevention of spreading of this bacterium in hospital environments (Quickel et al., 1971). Biosynexus Inc. is involved in development of protein-based pharmaceutical products aiming to ameliorate staphylococcal infections in infants, including lysostaphin. To provide a suitable production system for this selective antimicrobial enzyme, the encoding *lss* gene was cloned under control of the P*_nisA_* promoter and introduced in the expression host-strain NZ3900, allowing food-grade, lactose-based plasmid selection and induction of lysostaphin production through the NICE system (Mierau and Kleerebezem, 2005; Mierau et al., 2005). Following optimization of lysostaphin production at 1 L scale it was shown to be readily scalable to 300 and 3000 L production facilities. In addition, the protein could be purified from the lactococcal culture supernatant via straightforward and industrially scalable chromatography procedures (Mierau et al., 2005). This example illustrates that *L. lactis* is a suitable host for the production and purification of relevant bioactive enzymes, in this case an anti-*Staphylococcus* agent. Purification of the compound of interest. avoids the consequences of releasing genetically modified *L. lactis* strains and is suitable for bioactive molecules that do not depend on the presence or co-modulatory effects of the production host in the application.

## Live-Biotherapeutics Using Engineered *L. lactis*

Intrexon develops so-called Actobiotics, a class of orally delivered biopharmaceuticals employing engineered and biologically contained *L. lactis* production hosts for the *in situ* production and delivery of various proteins and peptides in the oral and gastrointestinal tract of humans. The selection of the bioactives to be produced within this delivery system aim to provide therapies against oral, gastrointestinal, metabolic, allergic, and autoimmune diseases, focusing on bioactives with known pharmacological activity and that are supported by documented efficacy and safety information. The company’s ambition is strongly based on the landmark study that demonstrated that chemically induced colitis in mice could be effectively treated with *L. lactis* strains that produce interleukin-10 (IL-10) (Steidler et al., 2000). Later work illustrated that the *L. lactis* based intestinal IL-10 delivery could also contribute to reduction of food-induced anaphylaxis in mice models, which may support the prevention of IgE-type sensitization to common food allergens (Frossard et al., 2007). Likewise, IL-10 delivery combined with secretion of the type-1 diabetes (T1D) auto-antigen GAD65370-575 by the same *L. lactis* strain was effective in suppressing T1D in a preclinical NOD-mouse model (Robert and Steidler, 2014; Robert et al., 2014). Besides these IL-10 producing *L. lactis* strains and their derivatives, the same platform for production and delivery of bioactives is employed for a variety of other molecules intending to provide live lactococcal therapeutics for a range of human and animal diseases (Vandenbroucke et al., 2004; McLean et al., 2017). Moreover, some comparative work using similar IL-10 producing *Lactobacillus* strains has been reported and may expand or enhance the application potential of these concepts (Steidler, 2003). Besides the bioactive production and delivery technology, the Actobiotics platform is supported by effective strategies for the biological containment of genetically engineered bacteria as well as scalable production and processing procedures for the live biotherapeutic bacteria (Steidler, 2003). However, initial human trials in patients suffering from inflammatory bowel disease established the safety and biological containment of the IL-10 producing lactococcal delivery vehicle (Braat et al., 2006), but failed to deliver convincing clinical efficacy evidence for disease symptom reduction to date, illustrating that translation of the mouse-model outcomes to human applications still requires further investigation.

Aurealis Pharma is a pharmaceutical company that employs genetically modified *L. lactis* strains for the delivery of combinations of therapeutic proteins to diseased tissues. For example, *L. lactis* MG1363 was engineered to allow plasmid-based expression of three genes encoding human fibroblast growth factor 2 (FGF-2), interleukin 4 (IL-4), and colony stimulating growth factor 1 (CSF-1) that were selected based on their functional properties (Yun et al., 2010; Hume and MacDonald, 2012; Sica and Mantovani, 2012) and availability of clinical safety data. An animal study demonstrated the efficacy of the combination therapy provided by the three proteins and the *L. lactis* strain in a delayed wound healing model in diabetic mice^1^. Aurealis Pharma is currently producing a clinical grade product for a clinical phase 1 trial to build a safety dossier for the use of this live bacterial therapeutic in diabetic foot ulcer patients.

## Perspectives

This short review illustrates that significant advances have been made in understanding the mechanism of action of endogenous probiotic *Lactobacillus* effector molecules, and that heterologous production of therapeutic bioactives in engineered lactococci has reached technological maturity. The examples chosen employed in this review are actively pursued by commercial initiatives and involve either purified bioactives or the use of the engineered LAB as live biotherapeutic.

However, in both strategies, the translation of the health-beneficial effects toward reliable human application is lagging behind despite the consistent findings across the different layers of the translational pipeline progressing from molecular insight in molecular interactions involved toward their conservation in *in situ* human response. Lagging efficacy evidence may be largely due to human individuality in health and disease, which implies that careful consideration should be given to selecting the appropriate target population. This may require novel strategies toward subject stratification and responsiveness prediction to better select and treat human subjects that are likely to have a perceivable benefit from the response elicited by the bioactive supplied.

## Author Contributions

Both authors conceived the review, wrote it together and decided to submit it to this Research Topic.

## Conflict of Interest Statement

PB was employed by the company NIZO Food Research BV. Part of the work by PB and MK was funded by the BE-Basic Program. Neither employer, nor funding agency had a role in the preparation of the manuscript.

## Footnotes

1 http://ewma.conference2web.com/#users/153991
